# Short Carbon Nanotube-Based Delivery of mRNA for HIV-1 Vaccines

**DOI:** 10.3390/biom13071088

**Published:** 2023-07-07

**Authors:** Yang Xu, Tammy Ferguson, Kazuya Masuda, Mohammad Adnan Siddiqui, Kelsi Poole Smith, Olivia Vest, Brad Brooks, Ziyou Zhou, Judy Obliosca, Xiang-Peng Kong, Xunqing Jiang, Masahiro Yamashita, Tsuji Moriya, Christopher Tison

**Affiliations:** 1 Luna Labs USA, 706 Forest St. Suite A, Charlottesville, VA 22903, USA; 2 Aaron Diamond AIDS Research Center, Division of Infectious Diseases, Department of Medicine, Columbia University Irving Medical Center, New York, NY 10032, USA; 3 Department of Biochemistry and Molecular Pharmacology, NYU Grossman School of Medicine, New York, NY 10016, USA

**Keywords:** carbon nanotubes, HIV-1, vaccine, mRNA, nanodelivery

## Abstract

Developing a safe and effective preventive for HIV-1 remains the hope for controlling the global AIDS epidemic. Recently, mRNA vaccines have emerged as a promising alternative to conventional vaccine approaches, primarily due to their rapid development and potential for low-cost manufacture. Despite the advantages of mRNA vaccines, challenges remain, especially due to the adverse effects of the delivery vehicle and low delivery efficiency. As a result, Luna Labs is developing a short carbon nanotube-based delivery platform (NanoVac) that can co-deliver mRNA and HIV-1 glycoproteins to the immune system efficiently with negligible toxicity. Surface chemistries of NanoVac were optimized to guide antigen/mRNA loading density and presentation. Multiple formulations were engineered for compatibility with both intramuscular and intranasal administration. NanoVac candidates demonstrated immunogenicity in rabbits and generated human-derived humoral and cellular responses in humanized mice (HIS). Briefly, 33% of the HIV-1–infected HIS mice vaccinated with NanoVac–mRNA was cleared of virus infection by 8–weeks post-infection. Finally, NanoVac stabilized the loaded mRNA against degradation under refrigeration for at least three months, reducing the cold chain burden for vaccine deployment.

## 1. Introduction

Synthetic messenger ribonucleic acid (mRNA) vaccines have become a promising alternative to conventional vaccine approaches because they can be quickly designed, tested, and mass produced [1]. mRNA contains genetic information that encodes a specific protein of interest for expression by the organism [2]. The use of mRNA to express specific proteins is a promising vaccine approach that avoids many safety issues associated with viral- or DNA-based systems. For example, when using a DNA vaccine there is always a risk it can cause a permanent change to the cell’s natural DNA sequence [3]. Early studies using mRNA were difficult due to the relative instability of the molecule and the preponderance of mRNA degrading enzymes (RNAses) in the natural environment [2]. Advances in RNA stability enhancement using modified bases and configurations indicate the greater applicability of mRNA for the purposes of gene therapy, vaccinations, and intra-cellular therapeutics [4]. 

Human immunodeficiency virus (HIV) is one area where the potential for use of mRNA has been suggested [5]. Although many strategies have been attempted for HIV vaccine development, none has been successfully established. The big challenges for HIV vaccine development are due to the vast diversity of HIV-1 and the formation of latent reservoir in cells that makes it very difficult to clear the virus using antiretroviral or immune therapy [6]. Now, scientists aim to harness the power of mRNA technology to fight HIV [7]. mRNA–based vaccine approaches have several advantages compared to classic protein-based vaccines. They are effectively expressed in the cytoplasm of a cell and can be targeted for any of the biological processes requiring mRNA as well as antigen protein production via HIV-specific mRNA. mRNAs are also non-specific activators of the innate immune system acting via Toll-like receptors (TLR) [8]. Alternatively, extensive evidence indicates that permanent control of HIV is mediated not simply by antibodies but primarily by effective HIV-specific CD8+ T-cells. Induced HIV-specific CD8+ T-cell responses could limit both the transmission and establishment of persistent viral reservoirs [9]. As a result, a T-cell-based HIV prevention strategy combined with ongoing B-cell vaccine approaches will be the primary consideration for future vaccine development. Recent technological advances have now overcome some of the issues with HIV vaccination and the delivery of mRNA–based vaccines. For example, the lipid nanoparticle (LNP)–based delivery of mRNA against infectious diseases and several types of cancer has demonstrated encouraging results in both animal models and in human use [10]. 

Despite the advantages of the LNP delivery of mRNA vaccines, challenges remain, including how to increase the payload of mRNA within LNPs, how to ensure uptake by cells and efficient expression in the cytoplasm, how to reduce the LNP toxicity, and more. As a result, there is a necessity to formulate mRNA vaccines with effective delivery systems that have negligible toxicity but provide functional vaccination. In this research paper, we report on a short carbon nanotube-based novel delivery platform (NanoVac) [11] that can deliver or co-deliver a broad range of mRNAs and encoding HIV-1 glycoproteins/peptides to antigen-presenting cells of the immune system via either intramuscular (IM) or intranasal (IN) routes. The NanoVac delivered vaccine candidates demonstrate robust systemic and cellular immune responses and potentially target specific mucosal sites, such as vaginal tissue, and thus could induce frontline immunity at the site of HIV-1 entry. This could help prevent the establishment and dissemination of an infection.

The HIV-1 vaccine candidate, RV144, is the only Phase 2b/3 clinical vaccine trial to date to demonstrate modest but significant efficacy in preventing HIV infection [11]. Subsequent studies of specimens from RV144 volunteers indicated that the only primary, independent correlate of reduced risk was a robust level of non-neutralizing antibodies (Abs) binding to a recombinant protein containing the first and second variable regions (V1V2) of gp120, a domain in the envelope (Env) glycoprotein of HIV-1 [12]. These data demonstrated that a V1V2-targeting vaccine has advantages over the imprecise targeting of SIV/SHIV infections and over whole Env-based immunization regimens for inducing a more focused functional V2p– and V2i–specific Ab response. Based on these studies, we used V1V2 (ZM53)-2F5K [13] as the target immunogen for the NanoVac delivery of its encoding mRNA in this work.

## 2. Materials and Methods

### 2.1. Short Carbon Nanotube Preparation 

The process of synthesizing short carbon nanotubes (SCNT) was described in our previous publication and is briefly summarized here [13]. As such, purchased multiwalled carbon nanotubes (MWCNTs, SES Research, Houston, TX, USA) were suspended in concentrated H_2_SO_4_/HNO_3_ solution (3:1 *v*/*v*) and sonicated in a water bath for 16 h at 40 °C to obtain carboxyl functionalities on the surface. This process also “cuts” the CNTs into shorter lengths to obtain short carbon nanotubes (SCNTs). Purification and multiple filtration/separation steps were performed to obtain different size ranges of short MWCNTs. The size of CNTs collected from each purification stage was determined using scanning electron microscopy (SEM) and dynamic light scattering (DLS) methods.

### 2.2. Short Carbon Nanotube Surface Modification for HIV-1 Glycoprotein V1V2 Loading

For the surface modification of SCNTs, detailed methods are again provided in our prior publication [13]. Briefly, a short chain PEG linker (NH_2_-PEG-COOH, MW, 2 K, Sigma-Aldrich, St. Louis, MO, USA) was selected for conjugation through the EDC/Sulfo-NHS method. The EDC/NHS–activated SCNT was conjugated with a PEG linker. The resultant PEG-SCNT was again separated and purified using a centrifuge filter (molecular weight cutoff of 10 KDa, Millipore, Burlington, VT, USA) three times to remove excess PEG. PEG-SCNT products were dried under vacuum for analysis and conjugation with V1V2 proteins via an EDC/NHS reaction. Thermal Gravimetric Analysis (TGA) curves of PEG-SCNT and SCNT alone were used to confirm and quantify the coating of functionalities (linkers) on the SCNT surfaces. The size of CNT samples was measured using DLS and SEM to ensure the obtention of proper dimensional SCNTs. 

### 2.3. mRNA Production

The antigen V1V2 (ZM53)-2F5K [14] –encoded mRNA sequence was submitted to Tri-Link for production. To stabilize the mRNA, the 5′ and 3′ UTR elements flanking the coding sequence profoundly influenced the stability and translation of mRNA, both of which are critical concerns for vaccine production. This HIV V1V2 sequence was capped with CleanCap AG (a fully mature, Cap1 structure), phosphatase-treated to remove any 5′ triphosphates, which has a synthetic 5′ UTR with a strong Kozak sequence, murine alpha-globin 3′ UTR, and a 120 mer poly A tail. Further, all uridines were substituted with N1-methylpseudouridine (Ψ) to increase translation and stability.

### 2.4. Short Carbon Nanotube Surface Modification for mRNA Loading

To obtain SCNT–mRNA conjugates, varying MWs (600–25 K) of polyethyleneimine (PEI) were first conjugated on the SCNT surface using the EDC/Sulfo-NHS method. To prepare different loading amounts of mRNA on the SCNT surface, SCNT-PEI was first formulated with 1,2-distearoyl-sn-glycero-3-ethylphosphocholine (EPC, Avanti Polar Lipids, Alabaster, AL, USA) in a weight ratio of 1:2 for 1 h via water bath sonication. Excess PEI and EPC were removed by centrifugation. Aliquots of SCNT-PEI–EPC were then mixed with EGFP-mRNA (a model green fluorescent protein encoding mRNA) or V1V2 encoding mRNA at mRNA:CNT mass ratios of 10:1, 5:1, 1:1, 1:10; 1:20 via pipetting and then incubated at room temperature for 10 min before tests. 

### 2.5. Cell Uptake and Simulation of DC Cell Maturation

THP-1 (American Type Culture Collection, TIB-202, Manassas, VA, USA) –derived immature dendritic cells (DCs) were harvested and counted for the SCNT uptake assay; the SCNT and SCNT-modified antigen samples were prepared at twice the concentration desired for the final volume/concentration. Briefly, 250 µL was removed from each well containing cells and replaced using 250 µL of the prepared samples. Media containing GM-CSF (0.5 µg/mL), IL-4 (1 µg/mL), TNF-α (0.1 µg/mL), and ionomycin (200 ng/mL) to induce maturation were added to the wells containing the positive controls. The wells were inspected under the microscope at 5, 15, 30, 60 min, and then 18 h and 5 days for uptake and cell maturation. The cells containing the Cy3™ dye labeled SCNT antigen was observed using fluorescent microscopy (ZEISS Axio Vert. A1 inverted microscope, ZEN 2.6 lite Blue Edition, North Chesterfield, VA, USA) with a red filter (Cy3/TRITC; excitation, 545 nm/emission, 605 nm). On the fifth day after SCNT exposure, the presence or absence of the surface marker for mature dendritic cells, including CD-83, was tested using a FITC-labeled monoclonal antibody. The anti-CD-83 antibody was added in a 1:10 dilution per the manufacturer’s recommendation and incubated for 30 min at 37 °C with 5% CO_2_. The free antibody was removed via aspiration and the cells were washed twice with fresh complete media. After the second wash, the cells were added with the fresh media and inspected via fluorescent microscopy using a green filter (FTIC/Cy2; excitation, 470 nm/emission, 525 nm). Following the initial inspection for the presence of CD-83-positive cells, the samples containing dye were re-examined for the co-localization of the CD-83 label with the SCNT label, indicating that the SCNTs may aid in the maturation of mature dendritic cells from immature dendritic cells.

### 2.6. Electrophoretic Mobility Shift Assay for Evaluation of mRNA Binding on SCNT 

The ability of SCNTs to bind and form a complex with mRNA (at different mass ratio between 10:1–1:20 as indicated in the above section) was determined by a mobility shift assay. The total mRNA amount was held constant at 0.25 µg per sample. To each sample, 7.5 µL of 2× RNA dye was added. Each sample was brought up to a final volume of 15 µL with RNAse-free water. The samples were then heated to 70 °C for 5 min then chilled for 3 min. The samples were then loaded onto a 6% TBE-Urea gel and run with a 1% TBE buffer at 180 mV at ~120 mA for 50 min. The gels were washed 3× with diH_2_O, five minutes each. The gels were then stained using the SYBR safe stain (Invitrogen ca# S33102, Waltham, MA, USA) at a 1:10,000 dilution in diH_2_O for 2 h in the dark. The gels were then washed again 3× with diH_2_O, 5 min each, and finally imaged under a UV light box.

### 2.7. In Vitro Transfection

THP-1 cells (American Type Culture Collection, TIB-202, Manassas, VA, USA) were maintained in T75 tissue culture flasks with growth medium RPMI-1640 (ATCC#30-2001, Manassas, VA, USA) containing 10% fetal bovine Serum, and 0.05 mM 2-Mercaptoethanol for about 3–4 days. The flask was kept at 37 °C with 5% CO_2_ to an immature dendritic phenotype prior to experimentation. For plating, the cells were collected via centrifugation and counted using a hemacytometer. The needed number of cells were separated and adjusted to 200,000 cells/mL in induction media (growth media, via the addition of growth factors hIL-4 and hGM-CSF at a final concentration of 100 ng/mL of media). Briefly, 100 µL of the cells in the induction media were added to each well of a 96-well plate and were maintained at 37 °C with 5% CO_2_ with media changes as needed (every 2–3 days). Prior to transfection, SCNT: EGFP-mRNA in a mass ratio of 1:1, 5:1 and 10:1 was added to SCNT solutions, as well as positive control lipofectamine (Invitrogen, #L3000008, Waltham, MA, USA), and all were allowed to incubate for 10 min at room temperature. Then, SCNT–mRNA complex solutions were loaded into each well, in triplicate, to yield final SCNT concentration of 5, 25 and 50 µg/mL. The positive controls comprised 0.2 µL of lipofectamine and 0.5 µg of EGFP–mRNA per well. All wells were monitored at 48 h.

### 2.8. In Vitro Mucosal Penetration Test

To test mucosal penetration in vitro, Calu-3 cells (American Type Culture Collection, HTB-55, Manassas, VA, USA) were seeded onto the apical side of 12–well transwell inserts at 3.0 × 10^5^ viable cells/insert. Trans-epithelial electrical resistance (TEER) measurements were taken approximately every other day to confirm cell confluency and tight junction formation. After thirteen days in culture, average insert resistance was higher than 1000 ohms, indicating that tight junctions had formed. Testing was then initiated the next day. Formulations with or without EPC were compared for performance. SCNT-PEI samples with and without EPC were prepared and sonicated for 1 h at ambient temperature. After sonication, samples were diluted in a ratio of 1:10 in complete culture media (EMEM, 10% FBS, ATCC, Manassas, VA, USA) bringing their concentration to 100 μg/mL. Fluorescein isothiocyanate dextran (Dextran-FITC) is a 3–5 kDa marker used to measure tight junction permeability [11,13,15]. Additionally, Dextran-FITC was added to all samples except the only control media at a final concentration of 100 μg/mL. Before adding the samples, time 0 TEER measurements were taken for each well. The basal chamber of each well was replaced with 1 mL of fresh media, and 500 μL of each prepared sample solution along with media only and media with Dextran-FITC only–controls were added to the apical chamber of each insert in duplicate. Samples were then returned to an incubator (37 °C, 5.0% CO_2_) for 1 h. TEER measurements were taken again before removing the full 1 mL of the basal chamber media to measure the FITC fluorescence (excitation, 487/10 nm; emission, 528/10 nm) contained in each basal chamber. Wells were replaced with 1 mL of fresh media in the basal chamber before returning to the incubator. These steps were repeated at the 2, 4, 18, 24 and 48 h time points. A Dextran-FITC standard curve using complete culture media was created to estimate Dextran-FITC concentration amounts in the basal chamber media for all samples at each time point. TEER values were normalized based on their T = 0 values for each individual insert.

### 2.9. Stability Test for mRNA Payload

A pair of primers was successfully designed on V1V2 HIV-1 glycoprotein sequences to amplify the cDNA produced from any RNA present in each sample. The primers were tested using a cDNA made from pure V1V2 mRNA that act as the positive control for the qPCR assay. To begin the stability study, 10 μL aliquots containing 4 µg of mRNA (at 1.5 mg/mL) with or without NanoVac were prepared. For NanoVac–mRNA samples, NanoVac formulations (4 µg of mRNA: 4 µg of SCNT-PEI: 8 µg of EPC) were incubated for 15 min at room temperature. The Zeta potential was measured to confirm that the complex was stable before testing. Samples were prepared for 7 timepoints in triplicate and stored at 4 °C for the duration of the study. At each time point, 5 μL per replicate was examined via gel electrophoresis while the remaining 5 μL was used to prepare cDNA using RT2 Easy First Strand Kit (Qiagen, 330421, Germantown, MD, USA). The resulting cDNA reactions were then stored at −20 °C. This step was performed at Day 0, 1, 7, 14, 23 and 30, and at three months (Day 90). Once all timepoints were collected, these cDNA samples and a positive cDNA control were used to run a qPCR assay. cDNA samples were diluted in a ratio of 1:100 in nuclease-free water, and 1μL of this dilution was then added to each 20 μL qPCR reaction along with the qPCR master mix (NEB, M3003S) and the forward and reverse primers (0.25 µM each). For the sample dilutions, each replicate was measured in duplicate wells (6 wells total for each timepoint sample). The positive cDNA control was diluted 10-fold and 1 μL of each dilution was used as the template for the standard curve with each dilution measured in triplicate wells. The qPCR thermocycling program included an initial denaturation step at 95 °C for 60 s, followed by 40 cycles each containing a 95 °C denaturation step for 15 s, a 60 °C extension step for 30 s and a plate read step.

### 2.10. Rabbit Immunogenicity Studies

Female New Zealand white rabbits 6–8 weeks old (~2 kg) each received three boosters. A total of 5 female New Zealand white rabbits in each group were dosed with 50 µg of NanoVac–mRNA at a volume of 100 µL via intramuscular administration. The rabbits were immunized via NanoVac–mRNA at weeks 0, 2, and 4 with blood collected at 2 (pre-bleed), 4 and 8 weeks (the end point of the experiment). Serum samples were collected to analyze the kinetics of the antibody response. Serum was separated from whole blood and stored at −20 °C until assays were performed. Body fluids (vaginal washes) were collected post-mortem. These analyses were specifically developed to compare both the breadth and potency of the antibody responses induced by mRNA and the encoding protein. At the end of the study, animals were euthanized via non-inhaled barbiturate injection under anesthesia. For the ELISA assay, 96-well flat-bottom plates were coated overnight at 4 °C with a HIV-1 V1V2 (ZM53)-2F5K immunogen at 1 μg/mL. The plates were washed with PBS containing 0.05% Tween-20 (PBST), at pH 7.4. Non-specific binding sites were blocked with PBS containing 3% BSA for 1 h. The plates were then washed once with PBST and subsequently 100 µL of rabbit serum was added and incubated for 1 h. Serum samples were diluted in a ratio of 1:10 for antibody detection. After incubation, the plates were washed with PBST. The bound antibodies were detected by incubating the with goat anti-rabbit IgG conjugated with alkaline phosphatase diluted in a ratio of 1:2000 in PBS. Finally, the plates were washed with PBST, and the bound alkaline phosphatase enzyme activity was revealed by adding 100 µL/well of the substrate solution. Absorbance at 405 nm in each well was measured using a microplate reader.

### 2.11. Immunogenicity Studies Using HIS Mice

The immunogenicity of Luna Labs vaccine candidates was measured in humanized immune system (HIS) mice. NOD.Cg-B2m^tm1Unc^ Prkdc^scid^ Il2rg^tm1Wjl/SzJ^ (NSG-B2m) triple mutant mice, which combined the features of severe combined immune deficiency mutation with IL-2 receptor γ chain and β2-microglobulin (β2m) deficiencies, were purchased from The Jackson Laboratories. Two–weeks after the adeno-associated virus serotype 9 (AAV9)–mediated transfer of HLA-A2/DR4 and human cytokine genes (IL-3, Il-4, IL-6, IL-7, IL-15 and GM-CSF), the NSG-B2m KO mice (4-5 weeks old) received a sub-lethal dose (150 cGy) of X-ray irradiation to myeloablate the remaining murine immune cells present in NSG-B2m KO mice [16]. A few hours later, the NSG-B2m KO mice were engrafted intravenously with 1 × 10^5^ human hematopoietic stem cells (HSCs), which were purified by CD34+ beads (Miltenyi, San Diego, CA, USA) from human cord blood cells. Fifteen weeks after the HSC engraftment, the percentages of human CD45+ cell repopulation, as well as the reconstitution status of various human immune competent cells, in the peripheral blood of AAV9-transduced NSG-B2m KO mice were determined via a flow cytometric analysis. As shown in the Appendix A Table A1, more than 60% of PBMCs of all the HIS-A2/DR4 mice were human CD45+ cells with a similar proportion of human CD3+ T-cells, CD4+ T-cells, CD8+ T-cells and B-cells, compared to that in actual humans.

Briefly, the first set of experiments was performed for four groups (three formulations and one control group as a preliminary test). Briefly, 10 HIS mice (five male and five female) were split into four groups. In each group, mice were immunized with each NanoVac formulation two doses in 3-week intervals via intramuscular injection (IM) and 50 µg of immunogen per mouse was administrated. In the second set of experiments, 10 mice were used for the studies. The NanoVac formulation was administrated by either IM or IN routes for two doses at 3–week intervals. For IM administration, 50 µg of immunogen per mouse was injected into the thigh at a volume of 0.1 mL. For IN administration, 20 µL of the formulation (50 µg of immunogen) was reconstituted for intranasal instillation using a micropipette. Two–weeks after the last boost, sera were collected from immunized, as well as naïve mice, and the titers of mouse IgG against HIV glycoprotein were determined via ELISA. Briefly, the ELISA microplates (Thermo Scientific, Durham, NC, USA) were coated with 75 µL per well of HIV V1V2 protein at 20 µg/mL, which was diluted in a coating buffer (Thermo Scientific). The plates were left overnight at room temperature (RT) with a sealer covered on top. On the next day, the plates were washed 3 times with PBS, blocked by adding a blocking buffer (Thermo Scientific, Durham, NC, USA) at 200 µL/well and left for at least 2 h at RT. Meanwhile, the serum samples were prepared with planned dilutions on a separate non-ELISA, non-coated 96-well round-bottom plate. The serum samples were diluted with the blocking buffer. The duplicates were prepared for each sample and dilution. After blocking, the plates were washed twice with PBS. Then, 100 µL/well of samples in appropriate serial dilutions were added to plates and they were left for at least 90 min at RT. The plates were washed 4 times with PBS-0.05% Tween, 100 µL/well of a 1:5000 (diluted-in-PBS-Tween) secondary antibody (HRP conjugated goat anti-human IgG Fc fragment; Bethyl Lab Cat#: A80-104P) was added and the plates were left for at least 1 h at RT. The plates were washed 4 times with PBS-0.05% Tween and 3 times with PBS, 75 µL/well of TMB High Sensitivity Substrate at RT (BioLegend Cat#: 421501, San Diego, CA, USA) was added and the plates were left for 10 to 15 min in the dark. Briefly, 75 µL/well of 2.00 Normal sulfuric acid was then added to the plates at RT to stop the reaction. Finally, the plates were placed on a spectrophotometer plate reader and the optical density (OD) was measured at 450 nm. 

The spleens were harvested and splenocytes isolated, followed by IFN-γ ELISpot assay to determine the relative number of HIV glycoprotein-specific human T-cell response. The relative numbers of splenic HIV V1V2–specific, IFN-γ-secreting human CD8+ T-cells of Luna Labs’ vaccines-immunized HIS-A2/DR4 mice were determined by an ELISpot assay, using a human IFN-γ ELISpot^PRO^ kit (Mabtech AB, Stockholm, Sweden) and a HIV V1V2 protein. A single cell suspension of splenocytes was isolated from the spleens harvested from the HIS-A2/DR4 mice 12 days after the last immunization with vaccines. Then, 5 × 10^5^ splenocytes in 200 µL/well of culture medium (RPMI-1640 with 10% fetal bovine serum and antibiotics and 5 × 10^−5^ M 2-mercaptoethanol) were placed on each well of the 96-well ELISpot plates pre-coated with anti-human IFN-γ monoclonal antibody (1-D1K) and incubated with or without 20 μg/mL of HIV-V1V2 protein for 24 h at 37 °C in a CO_2_ incubator. On the next day, the ELISpot plates were washed 5 times with PBS, and 100 µL/well of horseradish peroxidase-conjugated anti-human IFN-γ detection monoclonal antibody (7-B6-HRP) (diluted 1:200 in filtered PBS containing 0.5% fetal calf serum according to the company’s instruction) was added and the plates were left for at least 2 h at RT. The ELISpot plates were then washed 5 times with PBS, 100 µL/well of the ready-to-use TMB substrate solution was added and the plates were left until distinct spots were developed (5–10 min at RT in the dark). To identify the number of IFN-γ-secreting CD8+ T-cells in each well, the mean number of spots (for duplicates) counted in the wells incubated with splenocytes in the presence of the peptide was subtracted by the mean number of spots (for duplicates) counted in the wells that were incubated with splenocytes only.

### 2.12. Safety Studies for SCNT Vaccine Candidates

Forty (40) male Sprague Dawley rats (8–10 weeks old, Charles River Laboratories, Wilmington, DE, USA) were housed individually in solid-bottom cages with aspen bedding for the duration of the study. The study was conducted following a minimum 72 h acclimation, body weights were recorded (224–249 g) and animals were assigned to 8 groups of 5 rats each, achieving equal mean group body weights to the extent possible. One or three doses of NanoVac at a dose level of 0–30 mg/kg were intramuscularly or intranasally administrated at a dose level of 0–20 mg/kg for rats on Day 1, 14 and 28. Clinical observations were conducted daily. Body weight was collected approximately weekly and at the time of necropsy. Groups 1–4 were euthanized with isoflurane on Day 2, approximately 24 h post-dose. Groups 5–8 were euthanized on Day 42. Terminal blood was collected and processed for hematology and clinical chemistry as described below. All blood samples contained K3EDTA as an anticoagulant and were analyzed. Heart, lungs, kidneys, liver and spleen were collected, weighed, and fixed in 10% neutral-buffered formalin. The brain was removed, weighed and discarded. Slides of fixed tissue were prepared, stained with standard hematoxylin and eosin and reviewed by a qualified veterinary pathologist. Data were collected on a Microsoft 365 Excel spreadsheet. Graphic interpretation of the data was performed using GraphPad Prism 9.0.0 (GraphPad Software, Boston, MA, USA). Statistical comparisons of body weight, organ weight, hematology, and biochemistry parameters to vehicle control were made using Kruskal–Wallis one-way analysis of variance followed by post hoc testing using Dunn’s multiple comparisons test. In all cases, *p* < 0.05 was considered statistically significant.

### 2.13. Efficacy Studies Using HIS Mice

NSG-B2m KO mice were first transduced with human genes encoding HLA-A2/DR4 and human cytokines (IL-3, Il-4, IL-6, IL-7, IL-15 and GM-CSF) by AAV9 vectors, followed by being sub-lethally irradiated and engrafted with HLA-matched HSCs, as described above. Fifteen weeks later, more than 60% of PBMCs were found to be human CD45+ leukocytes with the composition of human B-cells and CD4+ T-cells, and the CD4+ T-cell subsets were similar to those observed in humans. Two groups of vaccine candidates were used for efficacy studies. One group was the SCNT-mRNA final formulation, and the second group was MC3 lipid nanoparticle (LNP)–encapsulated mRNA prepared according to published methods [10], serving as a benchmark control. The LNP mRNA contained the same amount of immunogen (50 µg) for delivery as the NanoVac candidate did. LNP was synthesized using a microfluidic device [10]. A third group was the naïve group, with no sample vaccine formulation delivered. Groups of 6 HIS mice (3 male and 3 female) were immunized intramuscularly with each mRNA vaccine two times in 3-week intervals. Then, 3 weeks after the last immunization, groups of immunized as well as naïve HIS mice were challenged via the intravenous administration of 2 × 10^5^ infectious units of a CCR5-tropic HIV-1 variant that is based on the LAI strain but carries part of the env gene from the CCR5-tropic Bal strain [17]. To confirm whether or not the HIS mice were susceptible to HIV-1 infection, we monitored the viral load in the plasma and CD4+ T-cell count in the blood after infecting the animals [17].

## 3. Results

### 3.1. Short CNT Delivered Vaccine Formulation (NanoVac) Development

**Figure 1:**
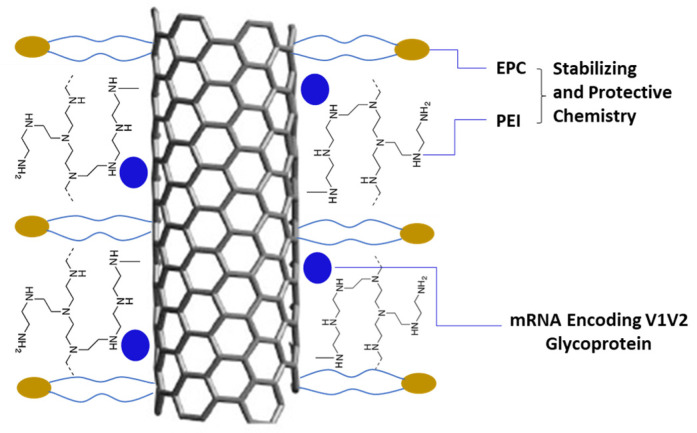
NanoVac–mRNA formulation schematic to demonstrate the surface chemistry of SCNT.

Raw CNTs were cut and separated using Luna Labs’ previously reported method to obtain short CNT (SCNT) at a range of 100–200 nm in length and 60–100 nm in diameter [13]. We then functionalized the surface of these SCNTs to load mRNA using cationic polymers. Cationic functionalities such as polyethyleneimine (PEI) and EPC (1-palmitoyl-2-oleoyl-sn-glycero-3-ethylphosphocholine chloride salt) were investigated in these tests. PEI demonstrated the highest transfection of cells in vitro (Section 3.2) in our studies. To further refine the formulation, we continued to use different-molecular-weight PEI (25 K, 10 K, 2 K, and 600, Table 1) for SCNT modifications. Linear and branched types of PEI were also compared. The surface charge of SCNT was significantly increased from negative 25.5 mV to positive charges after being coated with different MWs and types of PEI, as shown in Table 1. To further stabilize the mRNA using SCNT as the delivery vehicle, we included the lipid EPC which was in combination with PEI to immobilize on the SCNT surface (Figure 1). During optimization, we worked on the immobilization of EPC on SCNT-PEI in a ratio of 2:1 and 1:1. And then we tested the mRNA on EPC–SCNT-PEI in a ratio of 10:1, 5:1, 1:1, 1:10 and 1:20 using an mRNA binding assay to down-select the best candidates.

### 3.2. SCNT–mRNA In Vitro Evaluations

**Figure 2:**
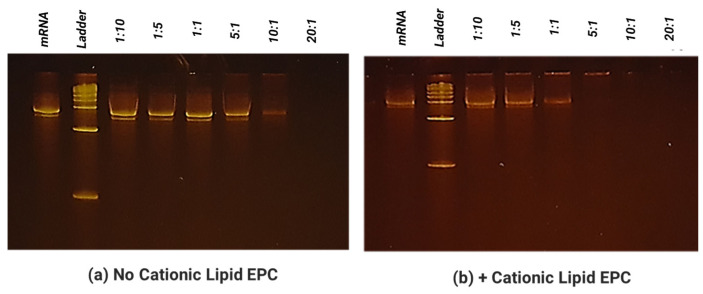
Binding capacity of SCNT-PEI towards mRNA without (**a**) lipid EPC and with (**b**) EPC.

**Figure 3:**
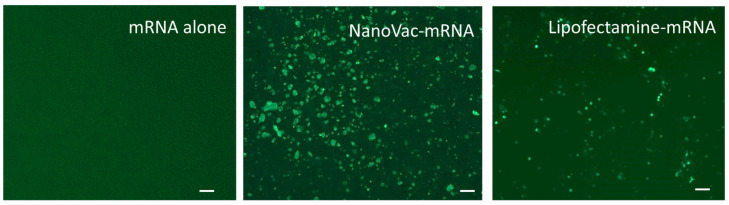
Fluorescence images of THP-1 cells transfected with GFP mRNA at 0.5 µg/mL for GFP production after 48 h. Lipofectamine was used as a positive control to compare with mRNA alone and NanoVac as the delivery vector. The scale bar indicates 100 µm.

We used an electrophoretic mobility shift assay to evaluate the mRNA binding capacity on SCNTs. The fluorescence dye SYBR green intercalated with free mRNAs, resulting in the visualization of the mRNA band under gel electrophoresis. The binding of SCNT to mRNAs resulted in a reduced intercalation of mRNAs by SYBR Green I, thus reducing the fluorescent signal. As shown in Figure 2, there was a gradual decrease in fluorescence intensity with an increasing SCNT to mRNA mass ratio. The migration of mRNA was completely inhibited when the mass ratio of SCNT to mRNA was between 5:1 and 20:1. These results indicated that SCNTs could bind and form a stable complex with mRNA at this optimized condition. It is also observed that without EPC in the formulation (Figure 2a), the binding capacity was much weaker than was the formulation with EPC added (Figure 2b). We used these conditions for the cell transfection test. In the cell transfection studies, the results presented in Figure 3 confirmed that the stable complex SCNT-PEI (25 K)–EPC: mRNA in a ratio of 1:1 showed the highest transfection compared to the positive control. The final average size of this complex was 268.8 nm and the Zeta potential was measured as +29.5 mV which further demonstrated that the NanoVac formulation was stable in solution.

Consequently, we used these above-mentioned ratios of NanoVac to mRNA for the in vitro transfection test. THP-1 cells were plated onto a 96-well plate for treatment with multiple ratios of NanoVac to mRNA. The results shown in Figure 3 confirmed that the stable complex NanoVac-mRNA demonstrated the highest transfection as compared to that of the positive Lipofectamine control or mRNA alone (no delivery vehicle for transfection). 

**Figure 4:**
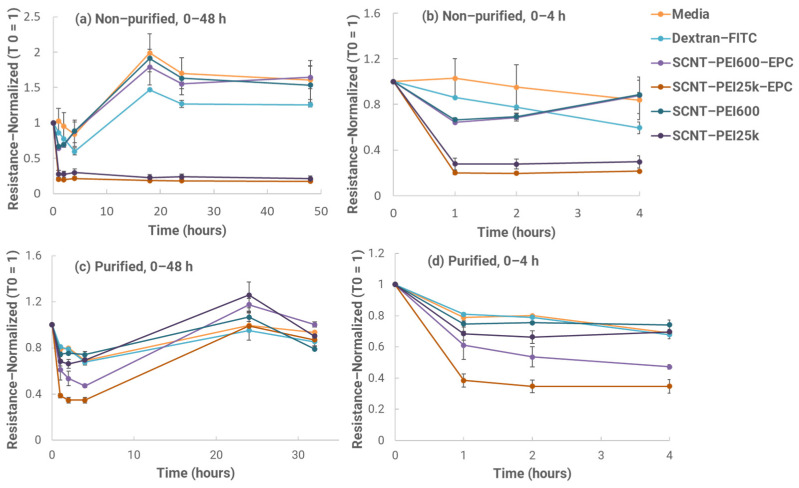
Resistance normalized over time during TEER test using Calu–3 cells after exposure to various SCNT formulations and controls at 0–32 h. Samples without (**a**,**b**) and with (**c**,**d**) purification are shown, with graphs on the right highlighting a response in the first 4 h of exposure.

To develop the intranasal version of our HIV-1 vaccine formulation, we used an in vitro mucosal penetration test model to evaluate SCNT formulation candidates with Calu-3 growth on a cell culture insert. We can use this setup to evaluate the penetration efficiency of vaccine formulations to select the best candidates. When establishing Calu-3 in Transwell inserts, cell polarization was measured for transepithelial electrical resistance. (TEER). Once Calu-3 liquid–covered culture (LCC) completely polarized and the TEER was no longer increasing (approximately two–three weeks of culture) at higher than 1000 Ω/cm^2^, Calu-3 LCC was ready for use as an in vitro model for characterizing epithelial cell responses to each formulation [13]. Two different molecular weights of PEI were selected (due to the surface charge, solubility, and transfection results) for penetration testing, including MW = 600 and 25 K. First, TEER resistance from cultures exposed to the SCNT-PEI 25 K with or without 1-palmitoyl-2-oleoyl-sn-glycero-3-ethylphosphocholine chloride salt (EPC) did not recover as the other two samples SCNT-PEI 600 did. Further, when observed under the microscope after 48 h, very few cells adhered to the surface of the insert, and most were floating in the media, indicating that the PEI 25 K concentration was too high and caused cell toxicity. As a result, extra steps would have been needed to be performed to remove the excess PEI 25 K that might have still been present in the samples to reduce the cytotoxicity of the excess PEI. Three repeated centrifugations/decants with washes were therefore performed and the samples were again measured in the insert to evaluate the TEER performance. Overall, the results indicated that cultures appeared to be recovered after 24 h, including the SCNT–PEI 25 K samples that had previously been too toxic for cell recovery. The slight drop in TEER values at 32 h can be attributed to the variance created when replacing the media. Cultures exposed to both sample types containing EPC showed lower initial TEER values up to 4 h (Figure 4) and generally higher Dextran–FITC amounts in the basal chamber with the SCNT-PEI 25 K–EPC–exposed cultures. This formulation was selected as the best candidate for animal studies.

### 3.3. Stability Test

**Figure 5:**
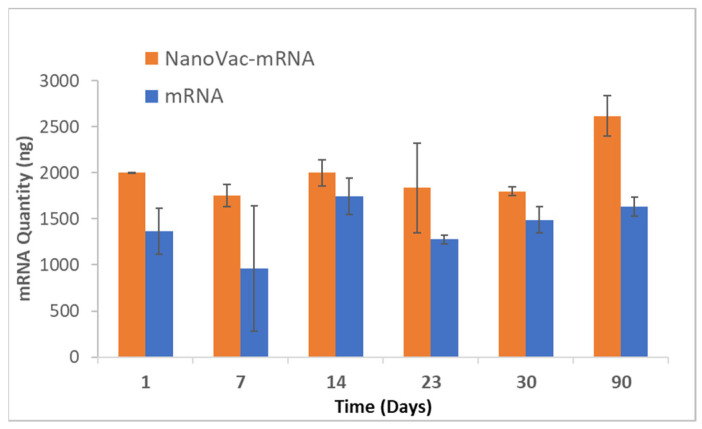
Long-term storage study of NanoVac–mRNA samples stored at 4 °C during a 3–month period. mRNA mass for each timepoint was determined using qPCR and quantified with a standard curve. The Day 90 timepoint was run on a separate qPCR assay, explaining the increase in signal over time.

The stability of mRNA was next evaluated using a reverse transcriptase–qPCR (RT–qPCR) method. We monitored the NanoVac–mRNA formulation under refrigerated conditions (4 °C, 65% RH) for up to 3–months. Data were then analyzed using a standard curve to calculate the relative quantity of mRNA from each sample, with the results shown in Figure 5. Based on these results, mRNA samples formulated with NanoVac were shown to be relatively stable over the 3–month period with minimal degradation observed. RNA gel was also used for the 3–month time point. It demonstrated that the mRNA bands were still bright and solid with minimal apparent degradation. This generally supports what was seen in the qPCR results. 

**Figure 6:**
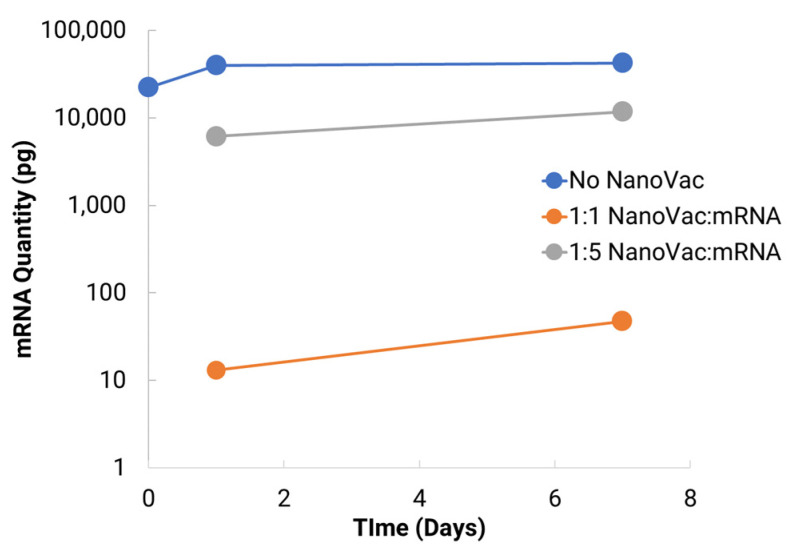
Seven–day storage study comparing the 1:1 and 1:5 NanoVac:mRNA ratios of the complexes. The amount of mRNA remained constant, and the amount of NanoVac was varied to reach the ratio of NanoVac:mRNA.

To help determine if the mRNA is detectable after binding to NanoVac, a smaller stability study was run comparing the effects of differing ratios of NanoVac to mRNA over a 7-day period. This study looked at two different ratios: 1:1 and 1:5 of NanoVac:mRNA, keeping the mRNA amount constant for both (2 μg) along with an additional sample containing no NanoVac. cDNA and qPCR reactions were run. These results showed that significantly more mRNA was detected with the 1:5 ratio compared to that detected with the 1:1 ratio (Figure 6). NanoVac can stabilize the mRNA on its surface without reducing the delivery quantity. Additionally, NanoVac:mRNA in a ratio of 1:1 (weight ratio) demonstrated much stronger binding to mRNA compared to the 1:5 ratio formulation.

### 3.4. Immunogenicity Studies Using a Rabbit Model

**Figure 7:**
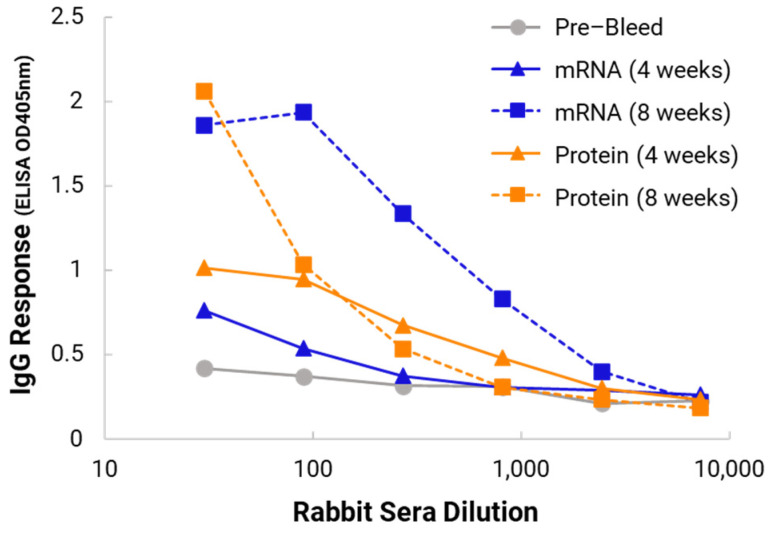
ELISA sera evaluation of IgG responses for NanoVac–mRNA-immunized rabbits against HIV-1 V1V2 (ZM53)-2F5K antigen. Results are provided at four (solid line) and eight (dotted line) weeks for both mRNA (blue) and protein (orange).

**Figure A1:**
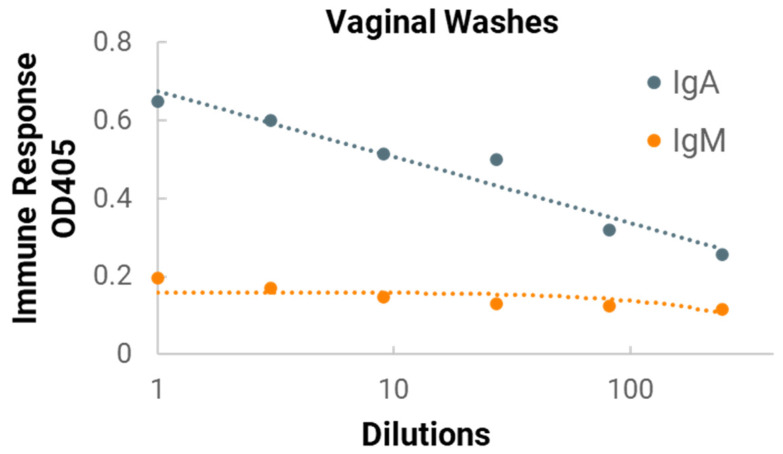
ELISA evaluation of IgA (blue) and IgM (orange) responses for NanoVac–mRNA vaccinated rabbits in vaginal washes from body fluids collected post–mortem.

Optimal NanoVac–mRNA candidates (NanoVac–mRNA and NanoVac–V1V2 protein) from in vitro studies were formulated to perform rabbit immunogenicity studies. As shown in Figure 7, we did not see a high immune response at 4 weeks. However, after 8 weeks and the third booster of the NanoVac–mRNA vaccine candidate, we observed a very high immune responses against the HIV-1 V1V2 (ZM53)-2F5K target antigen in the rabbit serum. When we compared the mRNA formulation with the administration of NanoVac–V1V2 protein (orange curves) as a control [13], we observed a higher and stronger IgG response for the mRNA candidate at 8 weeks (blue curves). Importantly, in the vaginal wash dilutions we also observed a relatively stronger IgA responses for the NanoVac formulation (Figure A1 in Appendix A) but not IgM. We then proceeded to a HIS mouse model for immunogenicity and efficacy studies using this formulation.

### 3.5. Immunogenicity Studies Using HIS Mice

The immunogenicity of Luna Labs vaccine candidates was measured in HIS mice. Confirmation of successful HIS mice production is provided in Appendix A Table A1. A first set of experiments was performed for four groups (three formulations and one control group). HIS mice in each group were immunized with each vaccine candidate two times in 3-week intervals via intramuscular injection. For IM administration, 50 µg of immunogen per mouse was injected. Three weeks after the second boost, sera were collected from immunized and naïve mice, and the titers of mouse IgG against HIV-1 glycoprotein were determined via ELISA. Finally, the spleen was harvested and splenocytes were isolated, followed by the performance of an IFN-γ ELISpot assay to determine the relative number of HIV-1 glycoprotein-specific human T-cell responses. NanoVac (SCNT-PEI (25 K)–EPC in a weight ratio of 1:2) was used as the mRNA delivery vector. Invivofectamine was used as a control for mRNA delivery. Sample preparation was performed according to protocols provided by Thermo Fisher. The delivery of protein V1V2 using NanoVac was also used as a control for comparison against the mRNA. 

When we measured humoral response against the HIV-1 V1V2 protein via ELISA, we found that groups of HIS-A2/DR4 mice receiving NanoVac–V1V2, and NanoVac–mRNA could result in a humoral response that resulted in significant human IgG titers against HIV-1 V1V2. In particular, mice immunized with the NanoVac–V1V2 group and mice immunized with the NanoVac–mRNA group induced more than 1/500 or 1/2500 IgG titers against HIV-1 V1V2, respectively. Since these HIS-A2/DR4 mice should have HLA-DR4–restricted human CD4+ T-cells, these T-cells were likely to help human B-cells produce class-switched human IgG upon receiving the NanoVac formulations that expressed HIV-1 glycoprotein V1V2. It would be interesting to see their human IgG subclasses and to determine if the sera react against HIV-1 in challenge studies. 

**Figure 8:**
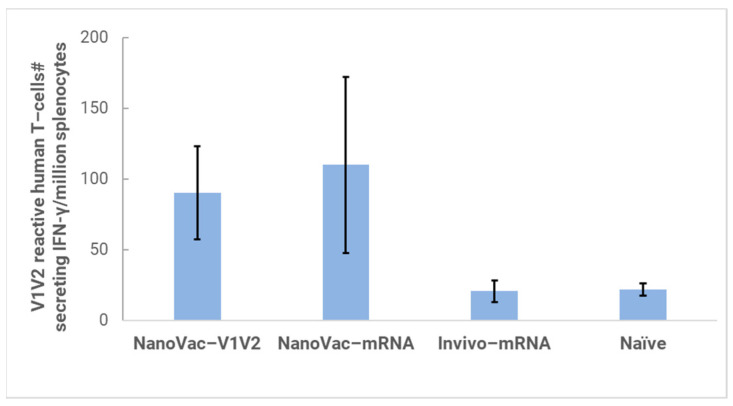
Human IFN-gamma ELISpot assay to evaluate HIS-A2/DR4 mice vaccinated with NanoVac formulations compared against Invivofectamine-delivered mRNA (invivo-mRNA) via IM administration and non-vaccinated mice as controls. Two mice were used for NanoVac–V1V2 and NanoVac–mRNA groups and three mice for invivofectamine and naïve control groups. Mice were dosed twice at 0 and 4 weeks, followed by sample analysis and termination after 8 weeks.

When we measured cellular response against the HIV-1 V1V2 protein via the ELISpot assay (Figure 8), we observed that groups of HIS-A2/DR4 mice vaccinated by NanoVac–V1V2, and NanoVac–mRNA formulations demonstrated an induced human T-cell response that resulted in a significant number of human T-cells secreting IFN-gamma in the presence of HIV-1 V1V2 protein in vitro. More than a hundred T-cells per one million splenocytes were collected from groups NanoVac–V1V2 and NanoVac–mRNA. They were found to secrete human IFN-gamma in response to HIV-1 V1V2, and the level of the T-cell response was closely correlated with the level of human IgG titers against HIV-1 V1V2 as determined via ELISA. Although the number of animals used in this study was small, the results still demonstrate that the NanoVac–V1V2 and NanoVac–mRNA vaccine candidates can induce both human-derived humoral and cellular responses against HIV-1 V1V2 antigen in HIS-A2/DR4 mice. 

**Figure 9:**
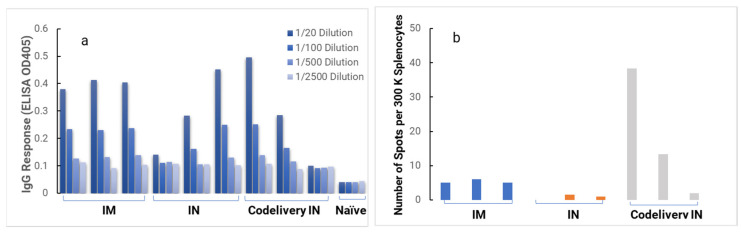
ELISA sera evaluation of IgG responses (**a**) and human IFN-gamma ELISpot (**b**) for each group of vaccine candidates, including NanoVac–mRNA via IM and IN routes and codelivery of mRNA and V1V2 antigen via IN route in HIS mice.

We next performed a second set of immunogenicity studies of NanoVac candidates, and a total of 10 HIS–A2/DR4 mice were used. Group 1, 2 and 3 mice received two doses of NanoVac formulations via intranasal (IN) or intramuscular (IM) injection in a 3-week interval. Group 4 was the control naïve mouse. Figure 9 shows the ELISA results from all three vaccine groups, with vaccinated HIS-A2/DR4 mice generating humoral responses that resulted in significant human IgG titers against HIV-1 V1V2. Group 1 mice immunized with NanoVac–mRNA (IM) possessed the highest IgG responses against HIV-1 V1V2 at dilutions of 1/500 or 1/2500, consistently. In the ELISpot assay, Group 1 formulation NanoVac–mRNA IM mice demonstrated a significant T-cell response. Since these HIS-A2/DR4 mice should have HLA–DR4–restricted human CD4+ T-cells, these T-cells likely helped human B-cells to produce class-switched human IgG upon receiving vaccines that express HIV-1 V1V2. Additionally, in the group three codelivery formulation administrated intranasally, one HIS mouse of showed the highest level of human T-cell response and the highest human IgG responses against V1V2 in ELISpot and ELISA, respectively. However, a moderate level of human IgG against V1V2 but no significant T-cell response was found in Group 2 mice. We hypothesize that this is because the NanoVac formulation performed as an adjuvant and played an important role in inducing intranasal absorption and T-cell responses. 

### 3.6. Efficacy Studies Using HIS Mice

In the efficacy studies, Luna Labs produced lipid nanoparticles (LNP (MC3)) as a benchmark delivery vehicle to compare with the NanoVac delivery platform, using a published method [18]. The weight ratio between the LNP (MC3) to mRNA is 10:1 for HIS mouse dosing. However, the weight ratio of NanoVac to mRNA in the NanoVac–mRNA group was 1:1, which significantly decreased the delivery vehicle used by approximately 10× while achieving the delivery of the same amount of mRNA. The plasma of each animal was isolated from the blood of vaccinated and unvaccinated HIS-CD4/B mice infected with an LAI variant of HIV-1 carrying the Env from the BaL strain, and the copy number of viral RNA was determined via qRT-PCR. The quantification of plasma viral loads was performed as described previously [19] with slight modification. Peripheral blood mononuclear cells (PBMCs) were isolated from vaccinated or unvaccinated HIV-1-infected HIS-CD4/B mice, and the percentage of human CD4+ T-cells among the total PBMCs was determined via flow cytometric analysis.

**Figure 10:**
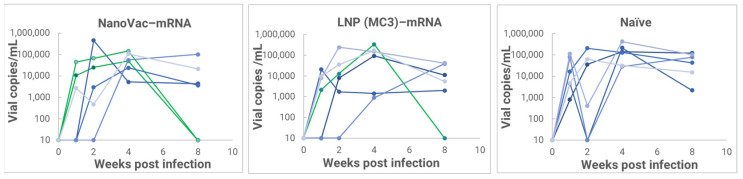
Plasma viral load in HIV-1-infected HIS-CD4/B mice following immunization with NanoVac–mRNA vaccine candidate and a LNP (MC3)–mRNA vaccine as the benchmark (each curve represents a mouse and green curves present the mice that were cleared out due to the vaccinations).

**Figure 11:**
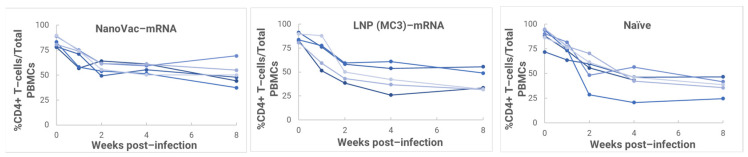
PBMCs isolated from IM–vaccinated or-unvaccinated HIV-1–infected HIS–CD4/B mice, and the percentage of human CD4+ T-cells among total PBMCs determined via FACS (each curve represents a mouse).

From the results, we found that inoculation with HIV-1 resulted in productive and persistent infection in all our HIS-CD4/B mice infected with HIV-1 with the viral load peaking at 3–4 weeks after the challenge (Figure 10) and that the percentage of CD4+ T-cells in the blood began to decrease gradually during the post-challenge (Figure 11). Without the administration of any vaccine group, animals demonstrated even more dramatically decreasing CD4+ T-cells. It is noteworthy that one of the HIV-1-infected mice previously vaccinated with the LNP (MC3)–mRNA vaccine and two of the HIV-1-infected mice previously vaccinated with the NanoVac–mRNA vaccine was cleared of virus infection by 8 weeks post-infection. Additionally, there was a trend that groups of mice vaccinated with either vaccine had a (significantly) lower virus load at 8 weeks post-infection. 

### 3.7. Biosafety Studies

**Figure 12:**
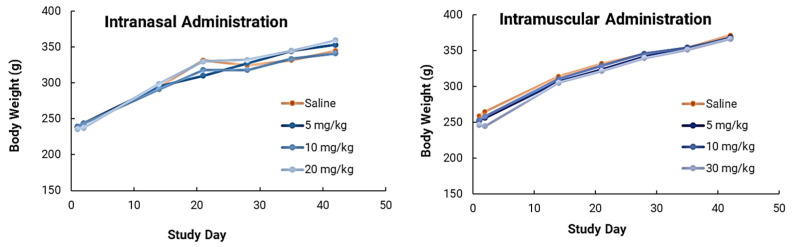
Body weight changes of rats during the experiment from Day 2–42. The NanoVac vehicle caused no adverse effects at up to 20 mg/kg (IN) or 30 mg/kg (IM) dosing.

**Figure A2:**
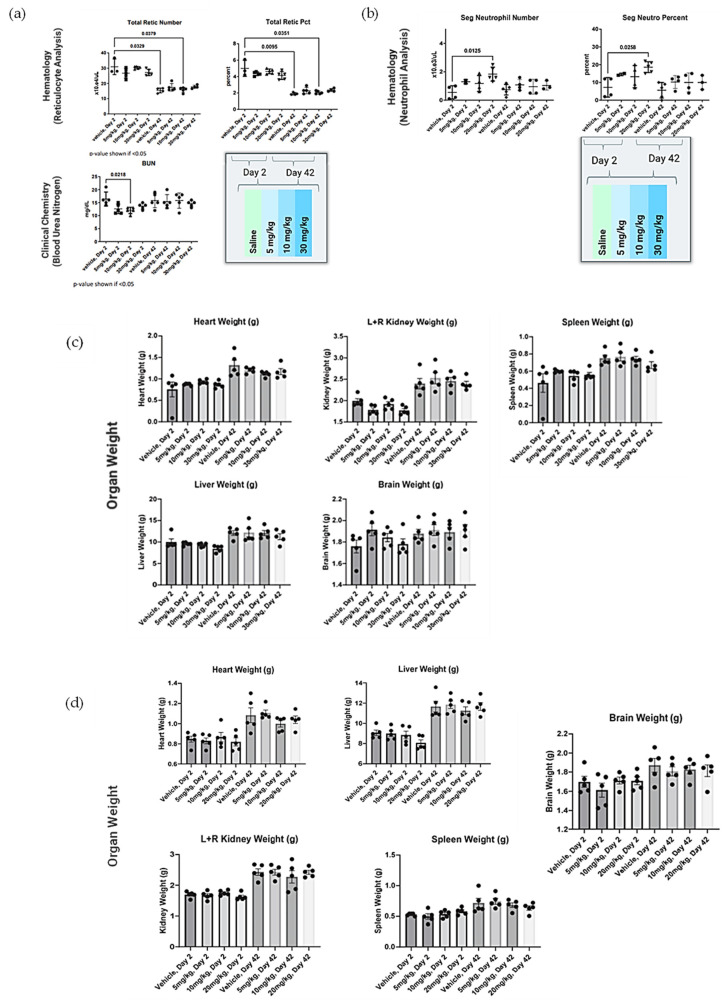
Hematology and clinical chemistry analysis for multiple parameters of terminal blood from each dose group of rats after IM (**a**) and IN (**b**) administration of short carbon nanotubes. Organ weight changes of rats during the experiment from Day 2 to 42 after the administration of NanoVac in different doses via IM administration (**c**) and IN administration (**d**).

We also performed toxicity studies of NanoVac in male Sprague Dawley rats via intranasal and intramuscular administration routes. The safety study was designed to investigate the one- and three- dose tolerability of NanoVac. For the intramuscular administrative route, short carbon nanotubes were clinically well-tolerated up to 30 mg/kg in male Sprague Dawley rats on Day 1, 14 and 28 and evaluated 24 h following the first dose or 2 weeks following the final dose (Figure 12). There were no biologically relevant effects on body weight gain, organ weights, hematology or clinical biochemistry parameters (Appendix A Figure A2). Adverse effects were limited to inflammation at the site of administration and local sequestration of SCNTs within macrophages and in lymphoid tissue. In intranasal administration, short carbon nanotubes were clinically well-tolerated at up to a 20 mg/kg dose when administered intranasally to male Sprague Dawley rats on Day 1, 14 and 28 and this was evaluated 24 h following the first dose or 2 weeks following the final dose. There were no biologically relevant effects on body weight gain, organ weights, hematology or clinical biochemistry parameters. SCNTs were visible microscopically around lung bronchi at 10 and 20 mg/kg. Adverse effects were limited to mild to moderate multifocal granulomatous pneumonia at the highest dosing of 20 mg/kg. Significant interalveolar neutrophils surrounded the regions of SCNTs and granulomatous pneumonia acutely but were absent by Day 42 post-dose. 

## 4. Discussion

The dimension of the as-produced nanotubes typically varies widely. They also have the tendency to form bundles and aggregates with varying shapes and sizes. To overcome this problem, Luna Labs first selected multiple-walled carbon nanotubes (MWCNTs) which have initial diameters of 60–100 nm and lengths of 1–2 µm as the starting materials. We then used concentrated H_2_SO_4_/HNO_3_ mixtures to cut the highly entangled long ropes of CNTs into narrow-size-distribution, short, open-ended non-entangled tubes. In this effort to specifically mimic the HIV-1 virus for subsequent vaccination, we refined the dimension of SCNTs as close to that of the HIV-1 virus as possible (120 nm in diameter). During the modification process, the MWCNTs become more biocompatible, biodegradable and stable in the aqueous solution [20] which is different than the case of unmodified CNTs as discussed previously [21]. We have demonstrated the gram-scale production of SCNT and achieved a narrow size distribution of high-purity short carbon nanotubes (SCNT) as the delivery vehicle. The purity of SCNT can reach greater than 99.64% as monitored using inductively coupled plasma optical emission spectroscopy (ICP-OES), as reported [13]. The purity of CNT is very important for potential product development, quality control and regulatory compliance for clinical use. We then successfully prepared NanoVac formulations as the vaccine candidates for mRNA delivery known as NanoVac–mRNA; antigen/protein delivery known as NanoVac–V1V2; or the codelivery of mRNA and V1V2 protein. Surface chemistries of the short multiwalled carbon nanotubes were also modified to guide antigen/mRNA density and presentation at the surface to better mimic the HIV-1 virion. This is a critical step for the use of this technology in vaccination efforts, where mimicking the virus and surface epitope presentation is critical to enhanced immunization [22]. As a result, the density of the antigen on the surface is a key factor. Luna Labs has demonstrated an optimized density for V1V2 protein conjugation and the control of the epitope orientation using small lipid-PEG as reported previously [13]. Recent technological advances have demonstrated multiple mRNA vaccine platforms that use the lipid nanoparticle (LNP) delivery of mRNA against infectious diseases. However, it cannot be modified for epitope presentation or the delivery of an mRNA-encoded protein with a controlled morphology. NanoVac shares its advantages with those of carbon-based materials which allows the better control of antigen presentation on the surfaces.

Two potential administration routes were explored using NanoVac as the vehicle, since the formulations were specifically designed/engineered for compatibility with both intramuscular and intranasal administration. To deliver mRNA into cells, we further modified/functionalized the surface of SCNT to achieve high loading capacity and delivery efficiency. We also engineered surface chemistry and functional design to stabilize loaded mRNA from degradation. One key challenge in creating nanodelivery mRNA complexes is that their transfection efficiency (TE) is adversely affected by serum, both in vitro and in vivo [23]. As a result, the design and formation of stable nanocomplexes of mRNA are critical, and the evaluation of their transfection efficiency for mRNA is required. Polyethyleneimine (PEI) has been demonstrated as an efficient gene delivery reagent both in vitro and in vivo [24]. Many studies using different types of PEI have shown relatively high efficiency in gene expression compared with other polymer vectors [25]. In our studies, after PEI was conjugated on the SCNT surface it enhanced cellular transfection efficiency. Additionally, the MW of PEI was found to be another key factor for transfection. We determined that using PEI at MWs of 25 K and 600 were the best conjugation candidates on the SCNT surface for transfection. Our data showed that the SCNT to PEI weight ratio of 1:1–1:5 was found to be the optimized condition of using the formulation. Additionally, to further block the surface negative charge of mRNA to form a protective layer, the optimized combinations of a multivalent lipid EPC and PEI on the SCNT surface stabilized mRNA and effectively achieved the maximum transfection delivery efficiency in vitro while also reducing vehicle cytotoxicity. The final formulation showed relatively high surface charges greater than 40 mV after centrifugation purification to remove the excess PEI and EPC polymer from the solution. Due to the positive charge density, it forms positively charged complexes with mRNA with high efficiency and provides efficient transfection and protection against nuclease-mediated degradation. Successful nonviral gene transfection currently requires compromises to achieve a useful level of transfection efficiency while minimizing toxicity. Thus, this purification step is critical to reduce the cytotoxicity concerns from these excess polymers observed in the TEER studies. The final formulation suspension was demonstrated to be very stable without any precipitation during injection/administration to animals. The size of the final formulation was 268.5 ± 117.0 nm with a surface charge of 29.5 ± 5.5 mV. 

**Figure 13:**
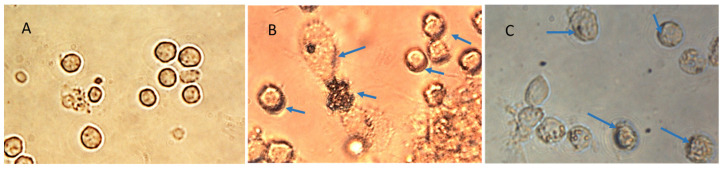
NanoVac uptaken by monocyte–derived immature dendritic cells (DCs) in a time–dependent manner. (**A**) Control cells, (**B**) 5 min uptake and (**C**) 18 h uptake. The blue arrow shows NanoVac uptake via micropinocytosis. The blue arrow points to the NanoVac uptake inside cells.

**Figure 14:**
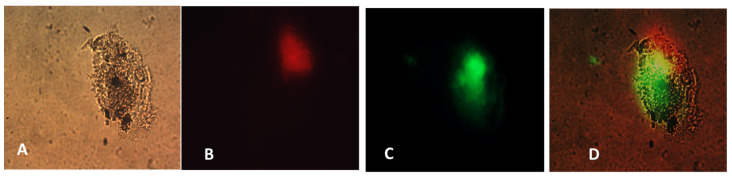
Fluorescence microscopy images of single cell. (**A**) Bright view; (**B**) Cy3 dye–labeled NanoVac uptake (red); (**C**) FTIC dye–labeled (green) mAbs for identification of CD83 expression; (**D**) merged images from (**A**–**C**).

The specifically designed and functional NanoVac–mRNA promoted rapid uptake within 5 min of exposure to antigen-presenting cells (APCs), such as dendritic cells (DCs). The uptake pattern is punctate, presenting numerous vesicles (Figure 13B) within the first 5 min. With a longer SCNT exposure time, the SCNTs began to be visible inside cells, suggesting that increasingly more SCNT–antigen conjugates were internalized (Figure 13C). The rapid and vesicular uptake is consistent with micropinocytosis, which is known to be an important mechanism of macromolecular antigen uptake in dendritic cells. As shown in Figure 13, we demonstrated that DCs internalized large amounts of NanoVac conjugates without compromising cellular integrity. Additionally, the chemistry of PEI plays a dual role to both bind nucleic acids and induce endosomal rupture via the so-called “proton-sponge” effect [26]. We also determined that the NanoVac activated DCs to express high levels of CD83, the cell surface marker for mature human DCs. DCs showed much greater responses to NanoVac compared to the antigen alone, likely due to the NanoVac shape and its functionality. NanoVac was able to fully activate the DC cells to become mature, as demonstrated via the CD83 expression and morphology (Figure 14), which indicated that the DCs matured and were able to promote MHC class II (major histocompatibility complex) and CD86 expression, critical to T-cell activation and the immune response. Due to the higher efficiency of NanoVac uptake into APCs compared to that of the antigen without a delivery vehicle, it will take significantly less time to complete the dosing, thus shortening the time to develop an effective antibody response. 

To increase translatability and RNA stability, along with in vivo delivery to activate innate immunity, we worked with TriLink to modify the mRNA encoding for V1V2. A 5′ cap, optimized 5′- and 3′-UTRs, and a coding sequence and poly(A)-tail modifications were introduced into the construction. We also modified nucleosides, using N1-methylpseudouridine (Ψ) to increase protein translation. Using NanoVac as the delivery vehicle, we further stabilized the mRNA from degradation during the storage under refrigerated conditions (4 °C) by tuning the chemistry on the SCNT surface. This was confirmed both by the qPCR results demonstrating recoverable mRNA, and an RNA gel at the 3-month time point that demonstrated that the mRNA bands were bright and solid with minimal apparent degradation. Interestingly, the quantity of mRNA recovered as monitored via qPCR appeared to increase slightly in quantity based on the qPCR results. It is inferred that over time, more of the mRNA bound to NanoVac was released and was able to be detected during the cDNA and qPCR reactions. This study looked at two different ratios—1:1 and 1:5—of NanoVac:mRNA, keeping the mRNA amount constant for both (2 μg) along with an additional sample containing no NanoVac. These results showed that significantly more mRNA was detected in the 1:5 ratio compared to that in the 1:1 ratio. We could potentially control the mRNA delivery via controlling the strength of binding and thus the releases from the surface. 

In safety studies for both intranasal and intramuscular administration, short carbon nanotubes were clinically well-tolerated to very high doses (20–30mg/kg) in male Sprague Dawley rats when evaluated after two weeks. There were no biologically relevant effects or clinical biochemistry parameter concerns. In ongoing research efforts, long-term safety studies will be performed because of the increased attention and safety concerns related to carbon nanotube use in human clinical trials. This issue is especially important because CNTs are extremely physically and chemically stable, and therefore may not be biodegradable. However, novel approaches for degrading CNTs have been developed and were implemented in this study. Luna Labs modified the SCNT surface through a strong oxidation reaction, which has previously been demonstrated to result in SCNT biodegradation in macrophages through a biological pathway [20]. This is an encouraging result, and, as demonstrated in our safety studies, adverse effects were limited to inflammation at the site of SCNT administration and local sequestration of the black pigment, likely SCNTs, within macrophages and in lymphoid tissue. Clinically, there have been promising published results of using CNT nanoparticles as a safe lymph node-harvesting tool [27], which indicates that SCNTs can be applied for human uses. Our results indicated that NanoVac is a safe platform that could be used for vaccine delivery. In the future, we will continue to expand toxicity studies, with specific focus on NanoVac intranasal formulations. We will use radiolabeled biodistribution studies to determine if, where and how much of the vaccine enters the nasal cavity, or even the brain, as this will help eliminate safety concerns with intranasal administration. This effort will support fully developed immunological profiles and a preclinical safety package before the performance of clinical studies. 

In the in vivo immunogenicity studies on rabbits, we compared the NanoVac–mRNA formulation and NanoVac–V1V2 formulation in eliciting immune responses. As shown in Figure 7, we did not see a high enough immune response during the earlier time point at 4 weeks for NanoVac–mRNA. However, after 8 weeks and the third booster of the NanoVac–mRNA vaccine, we observed significantly higher immune responses against the HIV-1 V1V2 (ZM53)-2F5K target antigen in the animal group than what we obtained from the protein V1V2 group. Even at a titer of 5000, we observed significant higher responses for the NanoVac–mRNA formulation compared to those with the administration of NanoVac–V1V2 protein as the control. It elicited two-fold higher IgG responses compared to those of the protein groups. Additionally, it is interesting to see IgA responses which were observed in the rabbit vaginal wash samples as shown in Figure A2 in Appendix A. 

Systemic immunization has generally been considered incapable of generating protective mucosal immune responses; however, cumulative data from recent studies suggest that some systemically administered vaccines are capable of eliciting mucosal immune responses, including the secretion of IgA antibodies [28]. The main hypothesis is that the antigen first diffuses from the IM immunization site to the regional draining lymph nodes, and from there is taken up by local APCs (such as DCs, B-cells and macrophages). These APC cells then migrate to mucosa-associated lymphoid tissue (MALT), such as Peyer’s patches (PPs) and nasopharynx-associated lymphoid tissue (NALT), where they activate CD4^+^ T-cells and B-cells [29]. The mechanism of this induction remains poorly understood. Though evidence points towards a mucosal response being generated using the NanoVac delivery vehicle, more comprehensive studies will be designed and performed in the future, including those comparing IgA response following IM and IN administration. A summary of the immune responses that have been evaluated from rabbit or mice studies to date, including those reported in our previous publication [13], is provided in Table A2, Appendix A. Combined, these results demonstrate that NanoVac is an effective adjuvant/delivery system for HIV-1 mRNA. Based on the rabbit results discussed above, we selected a HIS mouse model for further immunogenicity and efficacy studies to better interpret the results before moving on to NHP and human trials. 

In the HIS model, the produced HIS-CD4/B mice could mount functional human CD4+ T-cell and B-cell responses and were used for immunogenicity and challenge studies. In the HIS mice, more than 60% of PBMCs were found to be human CD45+ leukocytes with the composition of human B-cells and CD4+ T-cells, and the CD4+ T-cell subsets were similar to those observed in humans. In the HIS mouse model, Luna Labs clearly demonstrated that NanoVac-V1V2 and NanoVac–mRNA vaccine candidates were able to induce both human-derived humoral and cellular responses against HIV V1V2 antigen in HIS-A2/DR4 mice. Both the IN and IM administration of NanoVac elicited a robust IgG response in HIS mice models. The codelivery of protein and mRNA via the IN administration of HIS mice stimulated much higher T-cells responses than other individually administrated ones did either via the IM or IN route. In the efficacy studies, we found that inoculation with HIV-1 resulted in productive and persistent infection in all our HIS-CD4/B mice infected with Lai-BaL with the viral load peaking at 3–4 weeks after the challenge and that the percentage of CD4+ T-cells in the blood began to decrease gradually at 8 weeks post-challenge compared to that in uninfected HIS-CD4/B mice, as a control. It is noteworthy that two of the HIV-1-infected mice previously vaccinated with the NanoVac–mRNA vaccine was cleared of virus infection by 8 weeks post-infection. Using a benchmark LNP–mRNA vaccine, only one mouse was cleared of infection. Further, the NanoVac delivery vehicle decreased the lipid content delivered by 10x, which could explain the negligible concerns arising from toxicity studies. 

## 5. Conclusions

The current gold-standard mRNA delivery platform, MC3 lipid nanoparticles, was used as a benchmark in animal efficacy studies and we demonstrated that the NanoVac formulation outperformed this vehicle in the clearance of a HIV-1 viral load. The NanoVac formulations were also demonstrated to have stimulated both systemic and localized mucosal responses, and T-cell responses in rabbit and HIS mouse studies. Furthermore, we monitored storage stability (3–months) for NanoVac–mRNA under refrigerated conditions (4 °C and 65%RH) with no significant changes to recoverable mRNA. In toxicity studies for both intramuscular and intranasal administration, NanoVac was clinically well–tolerated at doses of up to 20–30 mg/kg in rats. These results indicated that NanoVac could safely deliver the chosen immunogens and induce a protective immune response in animal models. Based on our prior immunogenicity data and the confirmed effective response of our vaccine candidates in HIS mouse model, we demonstrated a major step in utilizing our mRNA delivery platform, NanoVac in either intranasal or intramuscular routes.

## 6. Patents

A provisional application for the patent “Surface modified carbon nanotubes and uses thereof” was submitted on 2 December 2022.

## Figures and Tables

**Table 1:** Characteristics of Zeta potentials for SCNT-PEI.

PEI Type	No PEI	Branched PEI				Linear PEI	
PEI Molecular Weight	N/A *	600	2 K	10 K	25 K	2.5 K	25 K
Zeta Potential (mV)	−49.9	+33.7	+41.5	+41.0	+ 48.6	+40.6	+34.8

* SCNT without conjugation with PEI.

## Data Availability

Data is contained within the article and Appendix A.

## Appendix A

**Table A1:** The percentages of human CD45+ cell repopulation, as well as the reconstitution status of various human immune competent cells, in the peripheral blood of AAV9-transduced NSG-B2m KO mice.

Experiment Name	Mouse ID	Vaccine	hCD45 #Events	hCD45 %Parent	mCD45 #Events	mCD45 %Parent	hCD3T #Events	hCD3T %Parent	CD8 T #Events	CD8 T % Parent	CD4 T #Events	CD4 T % Parent	CD19 B #Events	CD19 B % Parent	CD14 Mono #Events	CD14 Mono %Parent	CD56 NK #Events	CD56 NK % Parent
8-color phenotyping of 10 HIS-A2/DR4 HIS mice 3-31-2021	152	NanoVac-V1V2	94615	96.3	3590	3.7	56843	60.1	11948	21	44222	77.8	7558	8	758	5.4	8773	62.8
	153		77602	76.4	24019	23.6	35583	45.9	4905	13.8	30247	85	4908	6.3	408	2.4	10428	62.1
	123	NanoVac-mRNA	63869	65.3	33904	34.7	28122	44	13465	47.9	14172	50.4	3932	6.2	123	0.9	9285	64.8
	156		67185	64.4	37181	35.6	36594	54.5	9898	27	25819	70.6	4512	6.7	86	0.7	7809	67.2
	110	Invivo mRNA	77204	74.4	26605	25.6	31031	40.2	9028	29.1	21022	67.7	23920	31	577	5.7	4563	44.8
	112		64576	63.3	37395	36.7	23388	36.2	6316	27	16730	71.5	8264	12.8	173	1.2	11004	73.6
	113		75227	71.5	30034	28.5	28644	38.1	4728	16.5	23523	82.1	23157	30.8	434	4.1	5739	53.8
	118	None	39906	60.6	25895	39.4	16953	45.9	5793	34.2	10899	64.3	12345	33.4	240	6.7	2147	60.1
	119		59147	63.8	33507	36.2	25723	43.5	10600	41.2	14605	56.8	14970	25.3	69	0.8	5396	62.5
	120		46336	64.3	25677	35.7	22437	48.4	10118	45.1	11788	52.5	14003	30.2	36	0.8	2345	51

**Table A2:** Summary of immune response presence generated from different immunizations using NanoVac to deliver V1V2 protein (Pro) or V1V2 mRNA (mRNA), or the codelivery of both (Co). The presence of Pro, mRNA or Co in each cell correlates with a detectable response. Results presented in our previous publication are referenced [11] and data obtained from the current publication are bolded. “n/a” means not evaluated.

Administration Route	Immune Reponses	Rabbit	Mouse	HIS Mouse
IN	IgG	Pro [11]	Pro [11]	Pro, mRNA, Co
	IgA	Pro [11]	Pro [11]	n/a
IM	IgG	Pro, mRNA	n/a	Pro, mRNA, Co
	IgA	Pro, mRNA	n/a	n/a
